# The role of convalescent plasma and hyperimmune immunoglobulins in the COVID-19 pandemic, including implications for future preparedness

**DOI:** 10.3389/fimmu.2024.1448720

**Published:** 2024-09-09

**Authors:** Cynthia So-Osman, Thierry Burnouf, Arwa Z. Al-Riyami, Evan M. Bloch, Lise Estcourt, Ruchika Goel, Pierre Tiberghien, Marion Vermeulen, Silvano Wendel, Erica M. Wood

**Affiliations:** ^1^ Department Transfusion Medicine, Division Blood Bank, Sanquin Blood Supply Foundation, Amsterdam, Netherlands; ^2^ Department Hematology, Erasmus Medical Centre, Rotterdam, Netherlands; ^3^ Graduate Institute of Biomedical Materials and Tissue Engineering, College of Biomedical Engineering, Taipei Medical University, Taipei, Taiwan; ^4^ International PhD Program in Biomedical Engineering, College of Biomedical Engineering, Taipei Medical University, Taipei, Taiwan; ^5^ Department of Hematology, Sultan Qaboos University Hospital, Muscat, Oman; ^6^ Department of Pathology, Johns Hopkins University School of Medicine, Baltimore, MD, United States; ^7^ Radcliffe Department of Medicine, University of Oxford and National Health Service (NHS) Blood and Transplant, Oxford, United Kingdom; ^8^ Division of Hematology/Oncology, Simmons Cancer Institute at Southern Illinois University (SIU) School of Medicine, Springfield, IL, United States; ^9^ Dept Corporate Medical Affairs, Vitalant Corporate Medical Affairs, Scottsdale, AZ, United States; ^10^ Etablissement Français du Sang, La Plaine-St-Denis and Université de Franche-Comté, Besançon, France; ^11^ Department of Transfusion Medicine and Technical Services, The South African National Blood Service, Roodepoort, South Africa; ^12^ Dept Transfusion Medicine, Hospital Sírio-Libanês Blood Bank, São Paulo, Brazil; ^13^ Transfusion Research Unit, School of Public Health and Preventive Medicine, Monash University, Melbourne, VIC, Australia; ^14^ Department of Clinical Haematology, Monash Health, Melbourne, VIC, Australia

**Keywords:** COVID-19, SARS-CoV-2, convalescent plasma, scoping review, clinical use, plasma collection, adult, pediatric

## Abstract

**Introduction:**

When Coronavirus Disease-19 (COVID-19) struck the world in December 2019, initiatives started to investigate the efficacy of convalescent plasma, a readily available source of passive antibodies, collected from recovered patients as a therapeutic option. This was based on historical observational data from previous virus outbreaks.

**Methods:**

A scoping review was conducted on the efficacy and safety of convalescent plasma and hyperimmune immunoglobulins for COVID-19 treatment. This review included the latest Cochrane systematic review update on 30-day mortality and safety. We also covered use in pediatric and immunocompromised patients, as well as the logistic challenges faced in donor recruitment and plasma collection in general. Challenges for low resource countries were specifically highlighted.

**Results:**

A major challenge is the high donation frequency required from first-time donors to ensure a safe product, which minimizes the risk of transfusion-transmitted infectious. This is particularly difficult in low- and middle- income countries due to inadequate infrastructure and insufficient blood product supplies. High-certainty evidence indicates that convalescent plasma does not reduce mortality or significantly improve clinical outcomes in patients with moderate to severe COVID-19 infection. However, CCP may provide a viable treatment for patients unable to mount an endogenous immune response to SARS-CoV-2, based on mostly observational studies and subgroup data of published and ongoing randomized trials. Convalescent plasma has been shown to be safe in adults and children with COVID-19 infection. However, the efficacy in pediatric patients remains unclear.

**Discussion:**

Data on efficacy and safety of CCP are still underway in ongoing (randomized) studies and by reporting the challenges, limitations and successes encountered to-date, research gaps were identified to be addressed for the future.

**Conclusion:**

This experience serves as a valuable example for future pandemic preparedness, particularly when therapeutic options are limited, and vaccines are either being developed or ineffective due to underlying immunosuppression.

## Introduction

In December 2019, a new strain of coronavirus, named Severe Acute Respiratory Syndrome Coronavirus-2 (SARS-CoV-2) emerged in Wuhan, China, spurring a global health crisis (1). As convalescent plasma (CP) had been used in other infectious outbreaks (2), collection and transfusion of Coronavirus Disease-19 (COVID-19) convalescent plasma (CCP) was rapidly deployed globally to treat patients with COVID-19.

Use of CP to prevent and/or treat infectious diseases dates back to the mid-1880s, when serum therapies were employed to control diphtheria and tetanus (3, 4). CP is advantageous in infectious disease outbreaks when there is insufficient time to produce and disseminate other directed therapies, such as vaccines or hyperimmune immunoglobulins, extracted from convalescent plasma. CP can be mobilized rapidly, and historical accounts supported its safety and —potentially— its effectiveness against viral diseases like influenza, poliomyelitis, measles, and mumps (5–9). The Spanish influenza A (H1N1) of 1918 was the first viral pandemic in which a potential benefit of CP was reported (10–13). CP had since been used without notable adverse events or complications during other outbreaks such as West African Ebola epidemic (2013-2016) (14), avian influenza A (H5N1) and the influenza A (H1N1) pandemic in 2009 (15). In a prospective study, administration of CP to patients with severe H1N1 infection was associated with a significantly lower mortality and viral load as compared to controls (16). Pertinent to COVID-19, CP was used during the SARS-CoV outbreak in 2003 (17–21). In a study of patients with SARS-CoV in Hong Kong, the transfusion of CP with an antibody titer ≥1:160 before day 14 of illness was reported to improve outcomes (17). In a clinical trial, patients with confirmed Junin virus (the cause of Argentine Hemorrhagic Fever) who were transfused with high-titer CP within 8 days of symptom onset, had a significantly lower case-fatality rate compared to controls [1.1% vs 16.5%, respectively; (*p*<0.01)] (22). Both historic accounts of use, coupled with favorable reports of CCP early in the COVID-19 pandemic (23), provided a scientific and clinical rationale for passive polyclonal immune therapy to treat COVID-19.

**Figure 1:**
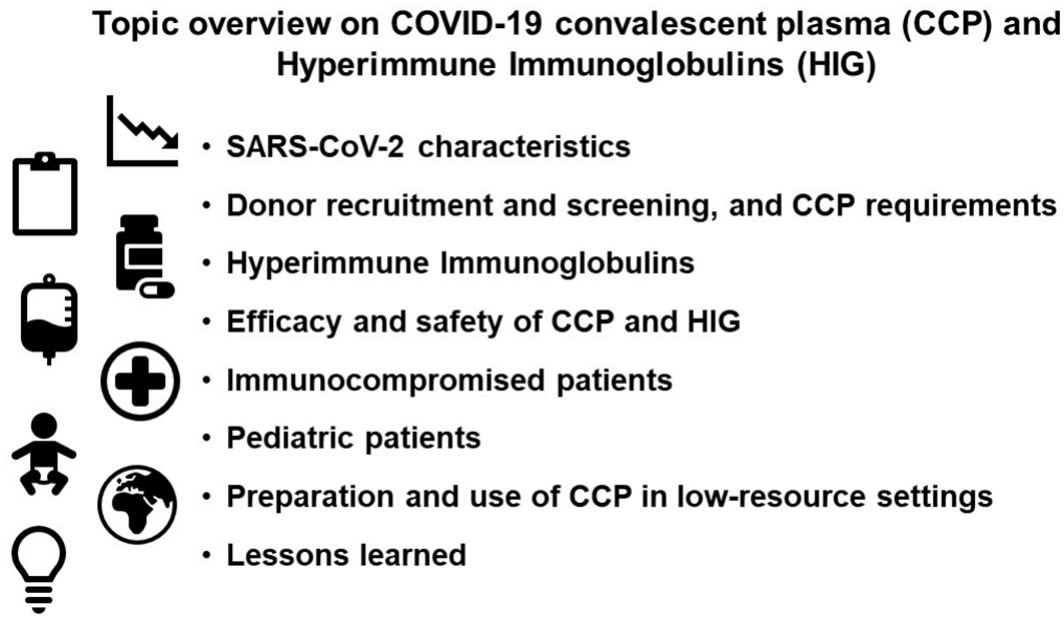
Topic overview of the use of convalescent plasma for COVID-19 (CCP) and Hyperimmune immunoglobulins (HIG).

Nonetheless, despite numerous reports of the use of CP, studies attesting to its benefit were overwhelmingly observational in design, limited by small sample sizes, and hindered by methodological limitations due to need of use on emergency grounds with the absence of other therapies. Moreover, the variability in how CP was qualified with regards to antibody titers, likely contributed to inconsistent dosing. Another limitation was the lack of uniformity in timing of administration. Despite consistent observations that CP needed to be administered early in relation to the onset of the infection for optimal benefit (22–24); several studies were conducted, highlighting, not unexpectedly, that CP was ineffective in unselected patients with advanced disease, such as influenza (25). Furthermore, the collective heterogeneity spanning the type of pathogen, study design and patient population accounted for uncertainty as to the therapeutic role of CP in the antimicrobial armamentarium. This review aims to provide an overview of the use of CCP and hyperimmune immunoglobulins for the treatment of SARS-CoV2 infected patients (**Figure 1**).

## SARS-CoV-2 characteristics

The SARS-CoV-2 virus is an enveloped, positive-sense, single-stranded RNA virus of the genus Betacoronavirus (26). It is a respiratory virus that relies on the receptor for angiotensin-converting enzyme 2 (ACE2) for entry into cells. The coronavirus virion is made up of a nucleocapsid (N) protein, membrane (M), envelope (E), and spike (S) proteins, which are structural proteins. The S protein of the viral particle is assembled as a homotrimer (three identical subunits) and is inserted in multiple copies into the membrane of the virion, giving it its crown-like appearance. The S protein of SARS-CoV-2 is cleaved into S1 and S2 subunits during its biosynthesis in the infected cells (27, 28). The S1 subunit binds ACE2 and the S2 subunit anchors the S protein to the membrane.

Transmission from animals to humans led SARS-CoV-2 to acquire a furin-cleavage site at the boundary of the S1 and S2 domains (27). This cleavage site was retained throughout the pandemic and acquired a D614G mutation, detected in Europe in February 2021 that compensated for the S-protein instability (29). Studies showed that the D614G mutation is associated with higher viral loads, enhancing binding of the virus spike to the ACE2 receptor, and increased infectivity (30).

In general, viruses escape immunity by mutating so that they avoid recognition by neutralizing antibodies (nAbs). As immune escape is necessary only in the presence of immune pressure, few escape mutations leading to variants of concern appeared in the early days of the pandemic, with lower dissemination and in the absence of vaccination. However, as the number of infected or vaccinated people increased, SARS-CoV-2 evolved to acquire S protein mutations as an escape route from nAbs, also potentially increasing the risks for immunocompromised patients (31). A number of variants of concern (VOC) have now been identified globally; the Alpha, Beta and Gamma VOC all emerged around the same time period between September and December 2020 in the UK, South Africa and Brazil, respectively, and had similar mutations in the receptor-binding domain (RBD) (29). These mutations were associated with higher viral loads leading to enhanced binding to the ACE2 receptor and increased infectivity (32–34). All variants exhibit decreased sensitivity to neutralization by immune plasma derived from convalescent patients with COVID-19 or vaccinated individuals *in vitro* (35–37). Later, the Delta variant and the Epsilon variant (California lineage) were identified (29). Studies using models to estimate population-level immune escape showed that the Beta and Delta VOC had limited reinfection rates in the population (38). However, this changed when the Omicron VOC was identified in South Africa in November 2021. Omicron, with 32 mutations in the spike protein, has rapidly spread globally. There have now been numerous subvariants of the Omicron variant with the currently predominant subvariant JN.1 and the latest identified subvariant BA.2.87.1 (39). Since BA.1, the virus has shown significant immune evasion from vaccines and the serum of patients who have recovered (40, 41). Thus, the capacity of SARS-CoV-2 to generate variants is a potential impediment to an optimal efficacy of CCP collected from donors who recovered from infection with previous lineages of the virus (42–44).

## Donor recruitment and screening, and CP requirements

Recruiting CP donors during a pandemic is very challenging. First, a host of logistical considerations requires strategies differing from those typically applied to routine whole blood or plasma donors. Second, a specific set of donor eligibility and screening procedures has to be approved and implemented to optimize the safety of the collection procedure for the donor and to the operators of the collection center, as well as that of the CP product for the recipient (45). In that regard, early availability of guidelines from international transfusion organizations and health authorities, and early sharing of experiences, were vital in providing clear recommendations worldwide on optimal collection and testing procedures of CCP (46–48). Third, early in the pandemic, the factors motivating convalescent patients to become CP donors (altruism, relief, gratitude for having survived, etc.) while anticipated, had yet to be determined in this specific setting. Notwithstanding, donor trust and attention to donor safety and privacy concerns are of paramount importance in a successful CCP program (49).

Once a CP donor has been effectively recruited, repeated donations, particularly at the early stage after convalescence, when nAbs are still at their peak levels (50), should be encouraged. However, with new VOCs described with recognized immune evasion capacity, periodic revisions are needed to reflect the predominant infectious variant and whether previous collected CCPs (mainly from the first and second COVID-19 surge) are still capable of adequate neutralization. Current advice recommends that newly CCP collections from recent infected and/or vaccinated donors should replace old CCP units remaining in stock (43).

CP donors are different from typical community blood donors in that they have recently recovered (in some cases have been recently hospitalized) and, may be donating for the first time. First-time donors usually present more risk factors than repeated donors (51), leading to higher deferral rates to decrease the risk of transfusion-transmitted infectious diseases (TTID). Although there is no evidence that SARS-CoV-2 is transfusion-transmitted (52), all CCP donors must meet the same eligibility criteria as regular plasma donors and be screened for TTID, according to current national or international guidelines. CCP can also undergo pathogen-reduction, with no impact on neutralizing antibody (nAb) activity (53), and should be considered specifically in settings where infectious testing and quality systems are suboptimal.

What constitutes a minimum level of nAbs suitable for CCP donation remains controversial, as there is great variability both in total IgG and nAb levels in each CCP donation (54). According to regulatory guidances (55), only very high titer, selected super-immunized CCP donors, mainly from those who had recovered from previous infection, should be accepted for transfusion (56, 57). Vaccines are known to increase IgG avidity against wild SARS-CoV-2 type in previously infected donors (58). A hybrid immunity (naturally infected and/or vaccinated CP donors) is also associated with increased binding and cross-reactive neutralizing antibodies against the newest Omicron SARS-CoV-2 variant and subvariants (44, 57–59). Molecular SARS-CoV-2 tests (by reverse transcriptase polymerase chain reaction methods; RT-PCR) are not required for CCP selection. There is still important variation between SARS-CoV-2 sero-neutralization assays (e.g. targeting live vs pseudo-virus; use of wild type vs currently dominant variants of concern; viral neutralization vs plaque reduction tests), with testing for nAbs serving as a gold standard. In addition, there is a correlation between the level of binding (anti-spike or nucleocapsid) with neutralizing antibody titers (60–62). Finally, if CCP donors donate more frequently or have their donor eligibility criteria modified (e.g., lower hemoglobin cut-off requirements) additional medical measures should be in place to ensure donor safety during and after CCP donation.

## Hyperimmune immunoglobulins

Although CCP has by far been the major human polyclonal immune therapy, polyclonal human hyperimmune immunoglobulin (HIG) against SARS-CoV-2 can be fractionated from CCP plasma. CCP units used for fractionation into HIG should meet all general quality requirements of plasma for fractionation set by plasma fractionators and their regulatory authorities. Fractionators may have additional specifications for donor selection compared to that for CCP (such as specific exclusion criteria related to travel restrictions or other safety criteria for blood-borne pathogens) and minimum requirements for anti-SARS-CoV-2 antibody titer in each donation (63).

Plasma for fractionation into HIG is most often collected by apheresis. The collected plasma is pooled prior to manufacture of HIG to ensure batch-to-batch standardization; these plasma pools may reach 4000 liters or more, representing approximately 5000 donations, unless a pilot-scale production is initially implemented (when feasible). The fractionation process is similar to that used for licensed immune globulins (IGs), which includes pathogen reduction measures, and concentration of the SARS-CoV-2 antibodies at least five to ten-fold (63–65). Each batch undergoes a range of mandatory tests to ensure consistency, quality and safety (64). Advantages of HIG over CCP include the diversity and concentration of Abs, the consistency and small volume of the final product; however, a major disadvantage is the relatively long delay (6 to 9 months) required for its production when using current industrial fractionation practices (63). General points to consider in the production and quality requirements of human polyclonal HIG against SARS-CoV-2 have been published recently (63).

## Efficacy and safety of CCP and HIG

Efficacy and safety of CCP and HIG have been evaluated in large cohorts and analyzed in systematic reviews, performing meta-analyses of high-level evidence from randomized clinical trials. Living systematic reviews (LSRs) have been performed by the Cochrane Hematology group on convalescent plasma, HIG and monoclonal antibodies (66, 67). The latest LSR on CCP and HIG evaluated clinical studies up to November 8^th^ 2023, and March 31^st^, 2022, respectively. For both LSRs, the search strategy to identify completed and ongoing studies was performed using the World Health Organization (WHO) COVID-19 Global literature on coronavirus disease Research Database, MEDLINE, Embase, the Cochrane COVID-19 Study Register, and the Epistemonikos COVID-19 L*OVE Platform.

For the CCP LSR, only randomized controlled trials (RCTs) evaluating CCP were included, irrespective of disease severity, age, gender, or ethnicity. Excluded were studies encompassing populations with other coronavirus diseases [SARS or Middle East respiratory syndrome (MERS)]. In this review, 46 completed studies with 25,469 participants were included, of whom 12,218 received CCP. Most participants included within the trials were affected by wild type or alpha variants of COVID-19, if reported. A further 45 ongoing studies were identified evaluating CCP. This review concluded, with high certainty, that for individuals with moderate to severe disease, CCP did not reduce mortality and had little to no impact on clinical improvement or disease progression. However, a subgroup analysis of a total of 1999 inpatients without antibodies at baseline showed significantly less risk of progression to mechanical ventilation or death after receiving CCP compared to 1657 controls with antibodies receiving standard care or placebo (risk ratio (RR) 0.91; 95% CI 0.84-0.98), with a statistically significant subgroup effect (*p*=0.02; 2 studies; I² = 82.8%). For individuals with mild disease, CCP may reduce the risk of hospitalization or death in unvaccinated individuals. This was seen for trials that compared CCP with standard plasma (reduction from 73 per 1000 to 36 per 1000, 95% CI 23 to 55; 2 trials, 1595 participants; moderate certainty), but not for trials that compared CCP to standard care or placebo. The potential benefit for immunocompromised patients is addressed in more detail in the next section on patient subgroups.

There was no clear difference in serious adverse events (SAEs); however, consistency in reporting of clinical outcomes and adverse events (for both the intervention and control arm) in future trials would help comparisons. In **Table 1**, a gap analysis of the clinical evidence on efficacy and safety of CCP is summarized. The large majority of published studies have reported no evidence of an antibody-dependent enhancement (ADE) associated with CCP. However, infrequent occurrence of early transient pulmonary worsening after transfusion of CP has been reported in two studies (78, 79). In vitro, antibody-dependent Fc receptor-mediated SARS-CoV-2 infection of macrophages is associated with inflammation while ultimately inhibiting viral replication (80, 81). Recently, such early pulmonary worsening followed by reduced virus replication in lungs and improved infection outcome has been reported in SARS-CoV-2–infected hamsters treated with high-titer CCP (82).

**Table 1:** Gap analysis of scientific evidence on the efficacy and safety of CCP for COVID-19.

Objective	Identified knowledge gaps	Focus points
**Trial and study design**	-What is the best way to assess efficacy and safety of CCP? -What control arm treatment should be employed in designing randomized clinical trials? -What other treatment alternatives to CCP are available? -What supportive measures are available? -How should patients crossing-over trial arms be managed?	- Wherever possible, use of CCP should be within the context of a clinical trial, preferable a randomized controlled study, until or unless its efficacy and safety are established. If CCP is used outside a clinical trial, data should still be collected to gather experience and outcomes. -Standard care, saline placebo, standard plasma in case of CCP study, anti-viral agents, standard IG in case of HIG study [both standard plasma and IG should have been collected prior to the pandemic or with no detectable antibodies] -Effectiveness of CCP compared to other treatment alternatives (e.g., anti-virals) should be assessed whenever possible. - All patients should receive the best available supportive care, driven by evidence-based guidelines. -In the absence of current effective treatment options, consideration should be given to patients in control arms crossing over to CCP treatment arms once the primary outcome of the trial has been met.
**Patient eligibility**	- Who would benefit most from CCP treatment? -What is the role of monitored use of CCP, if any, outside a clinical trial? - Who would benefit from CCP prophylaxis? -Who may not benefit from CCP treatment?	*Clinical trials should:* -Define the disease settings to assess which patients will benefit the most from therapeutic and prophylactic CCP and/or when CCP is not beneficial or even harmful. -Define patient eligibility criteria if any for monitored use of CCP outside a clinical trial (e.g if high titer CCP to be used in elderly, immune-compromised or with underlying comorbidity) (68, 69) -Current limited evidence doesn’t support its use. Updated patient eligibility criteria will be defined as new evidence emerges in the literature. Still being investigated in clinical trials (70) -CCP is not recommended in immune-competent patients with moderate or severe disease (71, 72)
**CCP/nAB dose, frequency and timing of administration**	-What is the minimum acceptable nAB dose to be effective? - What is the optimal dose of CCP? Does CCP dose vary between clinical settings (e.g. disease severity, different patient groups)? -When should CCP be administered in the course of the disease? -What clinical criteria define the need for a repeat dose(s)?	*Clinical trials should:* -Define minimum nAB dose needed for efficacious treatment. -Assess optimum CCP dose in a range of disease severities, clinical settings, and viral variants. -Based on available evidence, high titer CCP is recommended early in the disease course (< 3-8 days) in mild disease not requiring hospitalization (68). -Define clinical criteria that allow (repeated) CCP administration.
**Parameter to assess response and outcome**	-What clinical and laboratory parameters should be used to monitor response? - What are the best clinical outcomes to measure and what morbidity end points should be assessed? -How outcomes are related to antibody characteristics and titer levels? -What confounders could impact patients’ outcomes?	- Use of routinely collected data as much as possible will reduce workload pressure on front-line staff caring for patients. -Use precisely defined and globally accepted objective disease severity definitions, morbidity endpoints to assess response to CCP transfusions and to allow comparison of studies (73). Studies need to be developed to assess QOL outcomes -Assess for CCP antibody characteristics and titer levels, and compare these with the laboratory and clinical response. -Assess and control for confounders that could impact patients’ outcomes.
**Adverse events**	- Is CCP use safe? When is CCP uniquely unsafe? -Is CCP transfusion associated with higher risks of adverse events compared to standard plasma? -What hemovigilance definitions should be used to characterize adverse events in transfused patients? -Can SARS-CoV-2 be transmitted by blood transfusion? -Can CCP transfusion induce ADE or exacerbate underlying coagulopathy? -Is pathogen reduction technology warranted to reduce TTID risk? - Are there any novel adverse events that occur with CCP?	-Current evidence supports safety of CCP (74). Monitor safety of CCP and define settings in which it should not be used. -Monitor patients for adverse events while on treatment. - Use internationally agreed definitions used for standard blood components to gather more information on the safety of CCP collection and its use (75, 76) -Current evidence suggests that SARS-CoV-2 is not transfusion transmissible (77) -Current studies suggest that CCP does not induce ADE. More evidence is required to assess if CCP can exacerbate underlying coagulopathy - Current evidence of lack of transfusion transmission doesn’t support PI of CCP products -Published safety data confirms safety of CCP when compared to other blood components (74)
**Application in pediatric and neonatal medicine**	- What would be the eligibility criteria for use of CCP in pediatrics and neonates? -What CCP dose to be used and how frequent? -What clinical outcome should be assessed?	*Clinical trials should:* -Define eligibility criteria for use of CCP in pediatrics and neonates. -Define CCP dose and frequency of administration. -Define pediatric-specific clinical outcome measures to be assessed, especially outcome measures that can be objectively measured including change in clinical severity and neutralizing Ab titers in children post CCP administration.
**Less resourced countries**	-How to determine if use of CCP is feasible in settings of limited resources? -Are there any international programs to facilitate access to CCP for patients in medical systems with limited resources?	-Perform risk assessments for the use of CCP ideally including an assessment of the safety of the country’s blood supply in domestic blood establishments -Develop (international) programs to facilitate access to CCP for patients in medical systems with limited resources.
**Ethical considerations**	- How to prioritize CCP use if limited supply or if competition exists on the existing inventory between clinical trials and compassionate use? - How to implement CCP in settings with challenges in providing sufficient blood supply in LMICs?	-Define a mechanism on how to meet demand with insufficient CCP supply. -Diversion of resources away from routine blood collections in LMICs need to be carefully assessed.

Identified knowledge gaps and focus points in the use of CCP for treating COVID-19 patients (adapted from ^100^).

COVID-19; Coronavirus disease 2019, SARS-CoV-2; Severe acute respiratory syndrome coronavirus 2; CCP; COVID-19 convalescent plasma, TACO; Transfusion-associated circulatory overload, TRALI; Transfusion-related acute lung injury, TTID; Transfusion-Transmitted infectious Disease, LMICs; Low- and middle-income countries.

Regarding the LSR of HIG studies, five published studies were identified for hospitalized patients with moderate-to-severe disease, three were human-derived HIG products. The largest RCT (579 participants: ITAC study) compared the effectiveness of high-dose HIG 0.4g/kg (up to 40g) to saline placebo (83). These few studies resulted in limited evidence to know whether HIG affects death from any cause. No data are available for asymptomatic people with COVID-19 or people with mild COVID-19. Regarding safety, the ITAC study showed that HIG has little to no impact on adverse events of any grade on day 1 (RR 0.98; 95% CI 0.81-1.18; low-certainty evidence) compared to placebo. However, patients receiving HIG may, for still unclear reasons, experience more grade 3 and grade 4 adverse events compared to placebo (RR 4.09; 95% CI 1.39-12.01; moderate-certainty evidence).

## Immunocompromised patients

Passive polyclonal immunotherapies may offer particular benefit to patients with preexisting immunosuppression; this patient group is not only at high risk of complications of COVID-19, but they may also lack robust responses to SARS-CoV-2 vaccines (84). Furthermore, the emergence of SARS-CoV-2 variants and their associated resistance to monoclonal antibodies put immunosuppressed patients at risk for severe COVID-19.

The Cochrane group has published LSRs and a rapid review of the literature on the use of CCP and HIG (66, 85–87). Among these, five RCTs have assessed CCP efficacy among hospitalized patients with preexisting immunosuppression (i.e. malignancy, solid organ transplant, chronic steroid use, use of B-cell depleting therapies) (71, 88). Of these, the REMAP-CAP trial had a pre-specified outcome of evaluating CCP efficacy in immunosuppressed patients (71). A meta-analysis of the subgroup of patients who were immunosuppressed at baseline suggested that CCP decreased mortality compared to standard of care or placebo. Based on these analyses, the Association for the Advancement of Blood and Biotherapies (AABB) has suggested “CCP transfusion in addition to the usual standard of care for hospitalized patients with pre-existing immunosuppression, as a weak recommendation with moderate certainty of evidence” (89).

The most recent version of this LSR, which is not yet published, includes additional evidence from the CORIPLASM study (78). In a pre-specified subgroup analysis from this trial, CCP was associated with reduced mortality compared to standard of care only in hospitalized COVID-19 patients with underlying immunosuppression, while not reaching statistical significance (hazard ratio 0.39; 95% CI 0.14- 1.10). Of note, two independent case-control studies with propensity score matching in COVID-19 patients with underlying immunosuppression also reported reduced mortality with CCP treatment (90, 91).

CCP has demonstrated persistent ability to sero-neutralize variants (92, 93), as well as scalability and versatility. CCP may therefore provide a reliable treatment option for patients unable to mount an endogenous immune response to SARS-CoV-2 and its variants.

## Pediatric patients

While children usually develop milder COVID-19 disease, some groups of children with COVID-19 may have a higher risk of disease progression in association with immunosuppression, lung disease and/or cardiovascular disease. A passive polyclonal therapy could also be considered in immune-deficient children, who are unable to adequately respond to vaccinations, or in situations or countries where COVID-19 vaccines in children, especially the youngest ones who represent a particularly vulnerable group, have not yet been approved (94, 95).

Case reports early in the pandemic suggested that CCP transfusion was safe in children (96). A case-series of 13 children with severe COVID-19 and underlying chronic disease reported CCP as a safe intervention and showing clinical improvement when used within a median of seven days from symptom onset (97). One published protocol (98) and three clinical trials included children and neonates (99–101). One trial evaluated the safety and pharmacokinetics of high-titer CCP (≥1:320) at a dose of five mL/kg (maximum volume of 500 mL) in high-risk children (one month-18 years) who were either exposed or infected with SARS-CoV-2 (100). Although no adverse events were reported with CCP, this trial, which enrolled 14 treated children, showed that CCP use in high-risk children achieved neutralizing capacity and may protect against severe disease, but it was unlikely to provide lasting protection (100). The RECOVERY trial, a randomized open-label trial that randomized patients to standard of care with or without high titer CCP, included 26 children (<18 years), demonstrating no significant difference in 28-day mortality or hospital discharge between the two groups (99). In Canada, the CONCOR-KIDS trial, a randomized, multi-center, open-label phase two clinical trial of the safety and efficacy of CCP for treatment of COVID-19 disease in hospitalized children withdrew its registration without enrolment (101). Another single-center prospective, open-label trial evaluated CCP safety, neutralizing antibody kinetics, and outcomes in 46 children and young adults with moderate/severe COVID-19 (April 2020-March 2021). CCP showed significant improvement in COVID-19 severity score and neutralizing antibody kinetics suggesting CCP is well tolerated in children and young adults, providing rapid and robust increased neutralizing antibodies. Most recently, a multi-institute experience of 95 children receiving CCP in the USA was published. Median total plasma dose administered and transfusion rates were 5.0 ml/kg and 2.6 ml/kg/h, respectively (102). No serious adverse events were reported. Severity scores decreased significantly 7 days after CCP transfusion or at discharge, and 94.4% children survived to hospital discharge (103).

In summary, the use of CCP in children with COVID-19 disease appears safe, yet the evidence to conclude its efficacy remains limited. While multi-institutional collaborative trials will be ideal to study CCP efficacy in children, given the significant challenges in protocol approvals and enrolment, the role of national and international registries should be stressed for future pandemics.

## Preparation and use of CCP in low-resource settings

High-income countries (HICs) have an established infrastructure of (nationally coordinated) blood establishments to screen donors, collect and test donations, and implement pathogen-reduction technology. Such an infrastructure ensures that plasma products, including CP, have a high safety profile. The major challenges in low- and middle- income countries (LMICs) are access to and affordability of safe and effective treatments. CCP can be collected in low-resource settings, drawing on existing infrastructure. However, the blood system in most LMICs is less advanced than in HICs, and both safety and sufficiency of the blood supply are lacking in many LMICs.

Passive human polyclonal therapies using locally-collected plasma is attractive to LMICs in times of outbreak (104), pending the potential availability of low-cost treatment and/or preventive strategies, such as direct acting antivirals, monoclonal therapies, and vaccines. Manufacture of polyclonal therapies, such as CP, relies on the blood collection system for collection and testing of blood and plasma donations. Areas of need span donor selection (which is challenged by a typically high proportion of first-time, paid and family replacement donors, which, in the absence of advanced testing strategies, confer higher TTID risk than volunteer non-remunerated donors), infrastructure (e.g. equipment and facilities) to collect, process and store blood, human capacity (i.e. lack of skilled phlebotomists and technologists), availability of laboratory-based donor testing, quality management systems and regulatory oversight (104).

Two elements deserve specific attention regarding production of passive immunotherapies in LMICs. First, infectious risk is substantially higher than in HICs given the described challenges. If plasma is to be transfused, improved measures are needed to ensure low risk of TTIDs. These include rigorous risk-based assessment of potential donors, with a view to defer those with socio-behavioral and/or medical risk factors for TTIDs, and robust laboratory-based testing for the major TTIDs (e.g. HIV, HBV, and HCV) (104). Second is the mode of collection: the majority of CCP in HICs has been collected using apheresis, which is high-cost and requires skilled trained nursing personnel. CCP can also be produced from whole blood collections, which are low-cost and readily available, even in austere settings (105). However, its production is limited by the frequency of donations possible.

There is already a blood deficit in LMICs (106, 107). Therefore, production of CCP in LMICs risks the unintended consequences of diverting resources away from routine blood collections (107). Specifically, there is already an inability to respond to transfusion clinical demand, largely due to suboptimal donor recruitment (108). Demand for, and efforts to supply, CCP could exacerbate that deficit, thus adversely affecting clinical care for a wider group of patients.

Donor recruitment in LMICs often relies on replacement or even paid donors, both of which are potential risk factors for TTID in the absence of stringent donation testing (109). In large part the infectious risk is ascribed to over-representation of first-time donors (108, 110). Coupled with a high background prevalence for the major TTIDs (e.g., HIV) in many LMICs, along with deficient testing practices (e.g., limited quality oversight, exclusive serological testing in high incidence areas), render blood transfusion in LMICs to be a much higher risk medical procedure than that in HICs. In short, while CCP has been well tolerated in HICs (111), its risk-benefit profile needs to be re-evaluated in a low-income setting where infectious risk is pervasive.

Optimal effect of CCP is contingent upon the early timing of its administration relative to symptom onset —ideally in an outpatient setting (68, 69) as well as the antibody content of the product (112). Both these elements are challenging to ensure in a low-resource setting. Given limited access to care in LMICs, patients are more likely to present late in their disease process. Even in HICs, outpatient transfusion has been difficult to implement given a host of logistical, regulatory and administrative considerations (113). Further, given that high titers of antibodies are necessary for optimal effect, there is a need to qualify CCP donors and units accordingly. This requires testing, which in turn relies on laboratory capacity, which is frequently lacking in LMICs (114). Supply chain and procurement of assays, reagent costs, equipment (if run on automated platforms), and skilled personnel with the necessary technical expertise are needed to enable timely validation and implementation. Point-of-care tests have been developed that could facilitate qualification of CCP in a low-resource setting (115).

## What we have learned and implications for future pandemics?

It is now clear that we need international standards for the collection, testing, provision, and use of CCP. These standards should include estimates of the nAb titer since that is the most relevant functional component of CCP. Standardized protocols, including for donor screening and selection, would help ensure that products are comparable across different practices and regulatory systems. Key stakeholders included the International Society for Blood Transfusion (ISBT), the AABB, and international plasma collection and fractionation organizations including the CoVIg-19 plasma alliance. A host of other groups, spanning the clinical trial consortia, regulatory agencies, and international organizations (e.g., WHO) that are also involved in oversight of blood transfusion practices and/or provide technical assistance are critical to these efforts.

On the patient side, uniformity of clinical study criteria in terms of patient selection and outcomes evaluation is also needed. By introducing a common clinical scoring system to define the severity of illness, the effect of the interventions could be more easily assessed and compared (116). Next to the gap analysis on the clinical use of CCP (**Table 1**), a summary of key messages (**Table 2**) is provided.

**Table 2:** Panel table: Key messages.

Virus characteristics:
• In general, viruses escape immunity by mutating so that they may eventually escape recognition by neutralizing antibodies (nAbs).
• The SARS-CoV-2 Omicron variant, with 32 mutations in the spike protein, has rapidly spread globally. It had been shown previously that 20 or more mutations were sufficient for large population immune escape.
Donor and product issues:
• Convalescent plasma donor recruitment during a pandemic is challenging, requiring different recruitment strategies
• Donor trust and attention to donor safety and privacy concerns is of paramount importance
• Despite being considered as special donors, CCP donors must be qualified as any regular donors and screened for TTID, according to current guidelines.
• Collection procedures require specific measures to protect operators and ensure dedicated testing and labelling of the CCP unit
• Polyclonal human hyperimmune globulin (HIG) can be fractionated from CCP as set by plasma fractionators and their regulatory authorities
• The polyclonal character of CCP and HIG may be an advantage over monoclonals when variants appear
• It is important to check whether previously collected CCPs are capable of adequate neutralization of the VOC. In practice, consider to replace old CCP units with newer CCP collections from recent infected and/or vaccinated donors
Clinical trials:
• There are 45 ongoing studies evaluating CCP. Overall, CCP for individuals with moderate to severe disease did not reduce mortality and had little to no impact on clinical improvement and worsening. It had probably little to no effect on SAEs.
• Subgroup analysis showed that CCP is associated with a reduced risk of mechanical ventilation or death in inpatients without detectable antibodies at baseline compared to controls.
• The results of the ongoing studies, including large platform trials, might resolve some of the uncertainties around convalescent plasma therapy for people with asymptomatic or mild disease and for certain subgroups.
• Studies should report clinical outcomes consistently.
• Studies should report adverse events consistently, both for the intervention and the control arm.
• Reporting of subgroups is needed in studies evaluating patients with any disease severity. In particular, subgroup data is needed for SARS-CoV-2 variants, for level of antibody titer in donors and different ethnicities.
• Evidence on efficacy and safety of HIG remains scarce, more data are being collected.
Specific subgroups:
• CCP may provide a reliable treatment option for patients unable to mount an endogenous immune response to SARS-CoV-2.
• The use of CCP in children with COVID-19 disease has been shown to be safe yet the evidence to conclude on its efficacy remains limited
Low resource countries:
• Preparation of safe CCP in low- and middle-income countries is feasible and is a valuable and accessible therapeutic option making use of local blood resources until alternative therapies become potentially available and affordable
• A capacity-building program is important to guarantee the suitable quality and safety of CCP prepared from whole blood or by apheresis without putting additional constraints on the standard blood collection system delivering vital blood components to hospitals
• The risk-benefit profile of transfusing CCP in LMIC should be carefully evaluated to limit the occurrence of adverse events
Lessons learned:
• International standards for the collection, testing, provision and use of CCP is of vital importance. These should likely include estimates of the nAb titer measured against an international standard as the gold standard of efficacy
• Uniformity in international guidelines is also very important, based on high level evidence
• Large, well designed platform trials can serve as a way to collect high level data in a collaborative manner and within a short time span
• Agreement and collaboration across the major professional organizations who include dedicated professionals on this topic is important.

It became clear with SARS-CoV-2 that, when a new highly infectious virus is emerging, it is essential to gain knowledge as quickly as possible on accumulating scientific evidence as well as practical (operational) experiences and possible limits in clinical studies (117). Whichever a new pandemic afflicts mankind, it is essential in the future to keep up with three main principles of immunotherapy: a) presence of specific antibody; b) its presence in sufficient amounts and c) used early in the course of the disease (118). Again, collaboration is essential and can be achieved by sharing donor selection and testing approaches, as well as study trial concepts and protocols. Clinical study groups can collaborate by sharing data from ongoing and/or completed clinical trials as pre-prints, on a shared database (119), as meta-analyses (120), or by joining forces on collaborative studies. The international platform trials RECOVERY (99) and REMAP-CAP (71) are good examples of this approach. Their established platform designs facilitated rapid inclusion of new domains, their broad inclusion criteria were pragmatic and permitted greater generalizability of findings, and their multicenter approach enabled rapid recruitment. Together with the randomized study designs, they were able to rapidly achieve high-quality results which were implemented in national and international guidelines as soon as available (121). The Cochrane LSRs and meta-analyses are also very valuable in summarizing available data in a systematic way and on a regular basis (66).

How can professional organizations help? For CP, the worldwide collaboration of the key stakeholders mentioned above, together with regional and national societies, to share information and experiences has been essential. In 2020, at the start of the pandemic, the ISBT scientific working group on CCP performed an analysis to identify the gaps in scientific knowledge on how CP could be used most efficiently (122). However, four years after the start of the SARS-CoV-2 pandemic, many gaps still need to be addressed. Recently, a new collaborative action has been launched aiming to expedite clinical trials, ethical approval, reliable testing infrastructure to identify safe and efficacious CP, and donation pipelines during similar future crises (123). We can do even better in the future as we reflect on the lessons of the CCP trials, and we will be ready for the next challenge (124).

## Conclusions

There have been many global questions, challenges, and successes of CCP and, to a lesser extent, HIG, regarding efficacy and safety during the course of the COVID-19 pandemic. CCP appears safe, but efficacy of this therapeutic was not demonstrated in unselected patients with moderate to severe disease. However, it is of interest that vulnerable, immunocompromised, patients, lacking antibody responses, may benefit from this readily available human resource. The evolutive polyclonal character of CCP makes it likely more beneficial compared to mAbs preparations, as evidenced by the failure of mAbs to withstand the pace of mutation, resulting in diminished efficacy against variants overtime. Additionally, due to their high costs, mAbs are of limited interest in LMICs. It also remains to be seen whether CCP is confirmed to be effective when transfused early in the disease course. The lessons learned from use of these passive human polyclonal immune therapies can serve as examples for future pandemic preparedness, when therapeutic options are lacking, and while vaccinations are not yet available or are ineffective because of underlying immunosuppression.
